# Genome-Wide Identification and Expression Profiling of Wnt Family Genes in the Silkworm, *Bombyx mori*

**DOI:** 10.3390/ijms20051221

**Published:** 2019-03-11

**Authors:** Xin Ding, Junxia Liu, Lu Zheng, Jiangbo Song, Niannian Li, Hai Hu, Xiaoling Tong, Fangyin Dai

**Affiliations:** State Key Laboratory of Silkworm Genome Biology, Key Laboratory of Sericultural Biology and Genetic Breeding, Ministry of Agriculture, College of Biotechnology, Southwest University, Chongqing 400715, China; dingx305@163.com (X.D.); liuxiaoge@swu.edu.cn (J.L.); zl3188@email.swu.edu.cn (L.Z.); sjb2008@email.swu.edu.cn (J.S.); 13193064200@163.com (N.L.); huhaiswu@163.com (H.H.)

**Keywords:** Lepidoptera, silkworm, Wnt, expression profiling, gene cluster

## Abstract

Wnt is a family of conserved glycoproteins that participate in a variety of important biological processes including embryo development, cell proliferation and differentiation, and tissue regeneration. The Wnt family is a metazoan novelty found in all animal phyla. Studies have revealed that the number of *Wnt* genes varies among species, presumably due to reproduction and loss of genes during evolution. However, a comprehensive inventory of *Wnt* genes in Lepidoptera is lacking. In this study, we identified the repertoire of *Wnt* genes in the silkworm and seven other species of Lepidoptera and obtained eight *Wnt* genes (*Wnt1*, *Wnt5*–*Wnt7*, *Wnt9*–*Wnt11*, and *WntA*) in each species. Four of these *Wnt* genes are clustered in two orientations (5′-*Wnt9*-*Wnt1*-*Wnt6*-*Wnt10*-3′ and 5′-*Wnt10*-*Wnt6*-*Wnt1*-*Wnt9*-3′) in both moths and butterflies. Transcript analysis of *Wnt* in silkworm embryonic stages showed that each *BmWnt* gene had a unique expression pattern during embryological development. Analysis of a larval stage revealed differential expression of *Wnt* family members in diverse tissues. Our study provides an overview of the *Wnt* family in Lepidoptera and will inspire further functional study of the *Wnt* genes in the silkworm.

## 1. Introduction

When Nusse et al. studied mouse breast tumors in 1982, they discovered a protein then known as *int-1* that transmits proliferation and differentiation signals between cells [1]. Later, it was found that *int-1* was a homologous gene to *wingless* in Drosophila [2] and that the fruit fly gene encodes 54% of the amino acids in the same sequence and has 23 cysteines in the same position. The genes were thus named the *Wnt* gene family [3].

Wnt proteins are a set of secreted glycoproteins that contain 350–400 amino acids and are rich in cysteine. The molecular function of the *Wnt* family is relatively conserved among different species, and the *Wnt* family has been extensively studied in fruit flies and mammals. The transduction of Wnt signaling mainly depends on three pathways: the canonical Wnt signaling pathway, the non-canonical planar cell polarity (PCP) pathway, and the non-canonical Wnt/Ca^2+^ pathway. The three patterns are not mutually exclusive [4]. In the canonical Wnt pathway, Wnt proteins (Wnt1, Wnt3a, Wnt8, and Wnt8b) bind to the transmembrane receptor Frizzled (Fzd), resulting in stable accumulation of β-catenin in the cytoplasm and incorporation into the nucleus. This is followed by binding to the transcription factor T-cell factor/lymphoid enhancing factor (LEF1/TCF) family, thereby activating the transcription of downstream target genes to regulate embryo development and axis establishment [5,6,7,8]. In the non-canonical Wnt/PCP pathway, when Wnt proteins (e.g., Wnt5a and Wnt11) bind to the receptor Fzd, signals are transmitted through Disheveled to trimeric G proteins, which then activate downstream target genes Jun N-terminal serine/threonine kinase (JNK) and Rho-associated kinase (Rock), regulating cytoskeletal actin and cell polarity [9,10,11]. The non-canonical Wnt/Ca^2+^ pathway is mainly mediated by receptor Fzd2, which induces intracellular Ca^2+^ release and stimulates Ca^2+^–calmodulin-dependent protein kinase II (CamKII) and protein kinase C (PKC), thus affecting cell adhesion and gene expression [12,13].

Although the molecular function of Wnt is highly conserved, the number of *Wnt* genes varies greatly among different species. For example, only 3 *Wnt* genes are present in the demosponge *Amphimedon queenslandica* [14], while 21 genes have been identified in another sponge, *Sycon ciliatum* [15]. The *Wnt* family is divided into 13 subfamilies, and whole-genome comparative studies in some species have indicated that the 13 *Wnt* subfamilies were present in the common ancestor of cnidarians and bilaterally symmetric animals [16,17,18]. However, large-scale analyses have revealed that several *Wnt* subfamilies have been lost in many species during evolution. For example, the gastropod *Patella vulgata* has lost 9 of the 13 subfamilies, preserving only *Wnt1*, *Wnt2*, *Wnt10*, and *WntA*. In contrast, another gastropod, *Lottia gigantean*, has only lost two subfamilies (*Wnt3* and *Wnt8*) [19,20]. Vertebrates have retained all subfamilies except *WntA* [18,19,21], while more *Wnt* genes have been lost in insects [22,23]. Therefore, identification and characterization of the *Wnt* family should help us to understand the evolution of diverse species.

*Wnt* genes have been identified in insects such as *Drosophila melanogaster*, *Tribolium castaneum*, *Acyrthosiphon pisum*, *Anopheles gambiae*, and *Apis mellifera* [22,23,24,25]. However, there has been no systematic analysis of the *Wnt* repertoire in Lepidoptera. *Bombyx mori* is a representative insect of Lepidoptera. The research concerning the *Wnt* gene family in the silkworm has mainly focused on *Wnt1*. *BmWnt1* has been shown to be involved in embryonic development and the formation of larval markings and protruding structures [26,27,28]. However, besides the *Wnt1* gene, there has been very little research on other *Wnt* genes in the silkworm. In this study, we identified the repertoire of *Wnt* genes in eight Lepidopteran insects and further analyzed the expression profiles of the genes in the silkworm. This study provides a basis for understanding the distribution of *Wnt* genes in Lepidoptera and is conducive to further study of the function of *Wnt* genes in the silkworm.

## 2. Results

### 2.1. The Wnt Gene Repertoire in the Silkworm

To identify *Wnt* genes in the silkworm, we used the Wnt1 sequence as the query which for the Hidden Markov Model (HMM) is PF00110 from the Pfam database (http://pfam.sanger.ac.uk/) [29]. We searched the silkworm protein dataset using the hmmsearch program in the HMMER3.0 package (http://hmmer.org/) [30]. Nine proteins were obtained after manually removing redundant sequences and verifying the Wnt1 domain using the Simple Modular Architecture Research Tool (SMART, Available online: http://smart.embl-heidelberg.de/) (Table 1). Then, we further confirmed the nine *Wnt* genes obtained by searching for them in the assembled RNA-seq database on silkbase (http://silkbase.ab.a.u-tokyo.ac.jp/cgi-bin/index.cgi) and found that *BGIBMGA006147* and *BGIBMGA006148* are actually two parts of the *BmWnt10* gene. Consequently, eight *BmWnt* genes were finally identified in the silkworm genome (Table 1).

**Figure 1:**
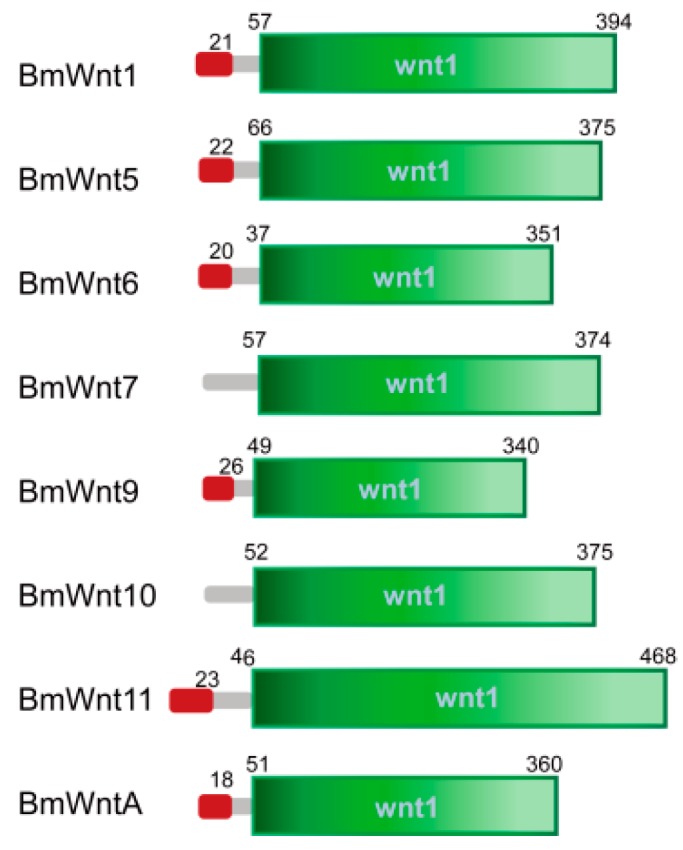
Structures of BmWnt proteins. Red indicates the signal peptide; grey indicates the non-specificity domain; and the green box shows the conserved Wnt1 domain. The numbers represent amino acid positions constituting the signal peptide and conserved Wnt1 domains.

The eight BmWnt proteins each possess between 340 and 468 amino acid residues. To analyze the protein structures of the BmWnt proteins, the functional domain was predicted by SMART. The results indicated that all the proteins contain a conserved Wnt1 domain (Figure 1). Alignment of the amino acid sequences of the eight BmWnt proteins showed that they shared 22 conserved cysteine residues, consistent with the characteristics of the Wnt family (Figure S1). Since the Wnt family belongs to the glycocrine proteins, we analyzed their signal peptides via the Signal 4.1 server (http://www.cbs.dtu.dk/services/SignalP/). The results revealed that signal peptides existed in six of the proteins while being absent in BmWnt7 and BmWnt10 (Figure 1; see Section 3 for further comments).

### 2.2. Phylogenetic Analysis of BmWnts

**Figure 2:**
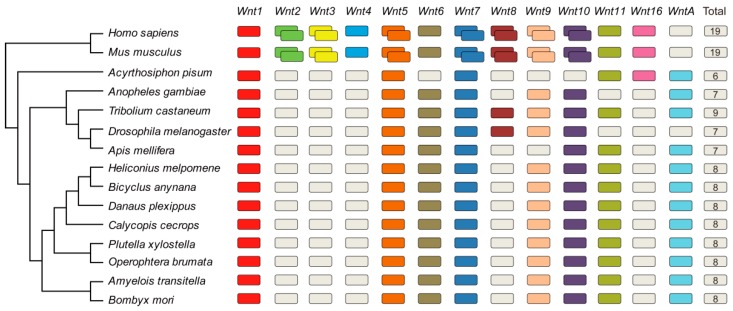
Summary of *Wnt* genes in 15 species. Differently colored boxes represent different subfamilies of *Wnt* genes; white boxes show the lost genes in the genome. Double boxes in the two top lines represent duplicated *Wnt* genes in mammals. The last column shows the total numbers of *Wnt* genes in different species.

To understand the phylogenetic relationships of BmWnt proteins, a neighbor-joining (N-J) evolutionary tree was constructed by MEGA 6.0 using the Wnt protein sequences of 14 other species comprising 2 mammals and 12 insects. The 2 mammals were chosen as the outgroup, and the 12 insects contained seven species of Lepidoptera (*Amyelois transitella*, *Bicyclus anynana*, *Calycopis cecrops*, *Danaus plexippus*, *Heliconius melpomene*, *Operophtera brumata*, and *Papilio xuthus*), two of Diptera (*Drosophila melanogaster* and *Anopheles gambiae*), and one each of Coleoptera (*Tribolium castaneum*), Hymenoptera (*Apis mellifera*), and Homoptera (*Acyrthosiphon pisum*). The Wnt proteins of the 14 species were identified according to the same method described above. There are a total of 19 *Wnt* genes in mammals, 7 in Diptera and Hymenoptera, 9 in Coleoptera, 6 in Homoptera, and 8 in Lepidoptera (Figure 2, Tables S1 and S2). We note that seven *Wnt* genes were identified in *Anopheles gambiae*, which is different from the six genes identified in previous reports. In mammals, there are two paralogous genes of *Wnt5*, *Wnt7*, *Wnt8*, *Wnt9*, and *Wnt10*, while only one paralogous gene was identified in insects. The mammals possess *Wnt2*, *Wnt3*, and *Wnt4* and have lost *WntA*, while the insects possess *WntA* (except for *Drosophila melanogaster*) and have lost *Wnt2*, *Wnt3*, and *Wnt4*.

**Figure 3:**
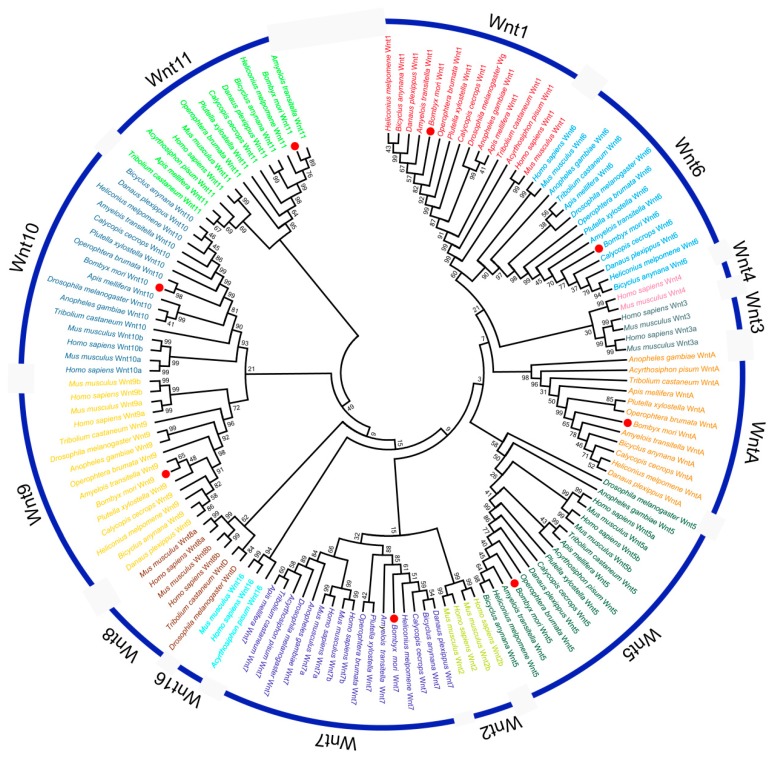
Phylogenetic tree of *Wnt* gene families. The *Wnt* genes are distinctly divided into 13 subfamilies shown as different colors. The eight *BmWnt* genes are marked with red dots.

The *Wnt* family is divided into 13 distinct subfamilies. Most clusters of orthologues were divided into two branches, a vertebrate branch and an arthropod branch, except for the *Wnt7* and *Wnt11* clusters. In the *Wnt7* and *Wnt11* clusters, genes in Lepidoptera separate into a cluster, while the others are divided into a vertebrate branch and an arthropod branch (Figure 3), suggesting that *Wnt7* and *Wnt11* in Lepidoptera showed different evolutionary dynamics.

### 2.3. The Conserved Wnt Gene Cluster

**Figure 4:**
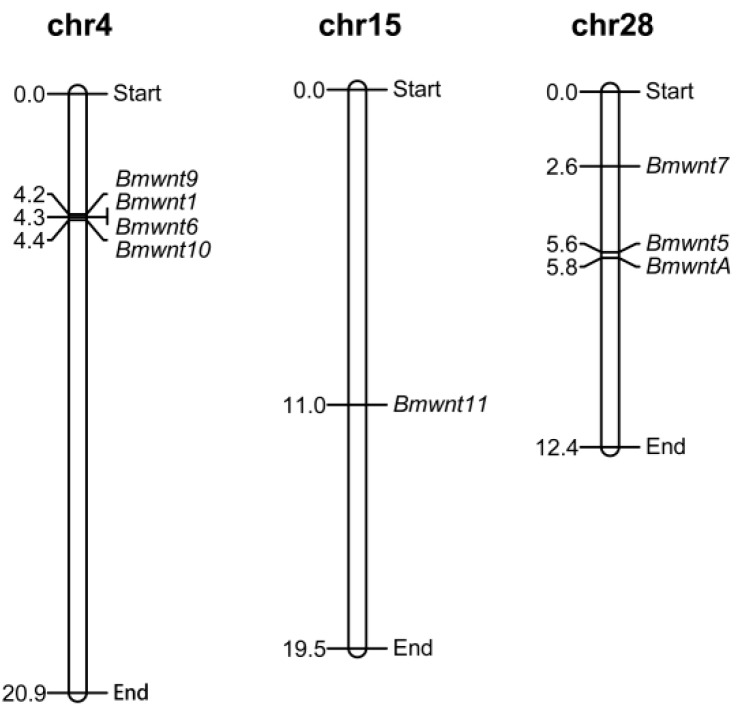
Map of the distribution of *BmWnt* genes on silkworm chromosomes. The corresponding numbers left of the chromosomes suggest the gene location of the chromosomes.

**Figure 5:**
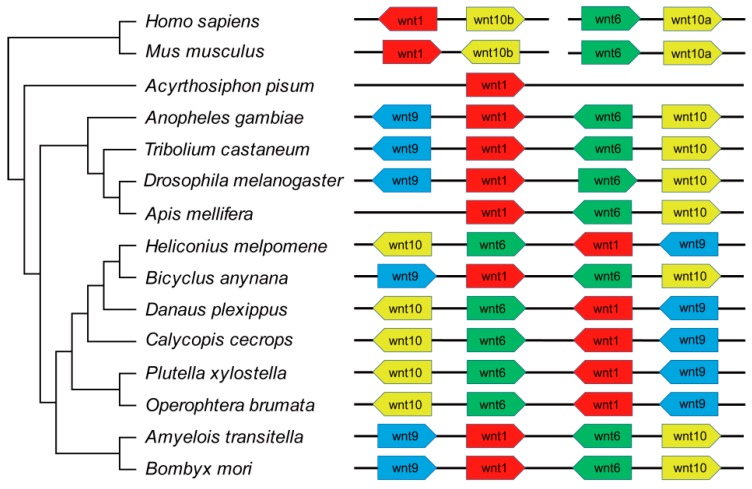
The conserved *Wnt* gene cluster in the 15 species. Paralogous genes are shown in the same color, and the orientation of genes is represented by the block arrows.

To determine the distribution of *BmWnt* genes on silkworm chromosomes, we mapped them based on their coordinates from the silkbase database. Four of the eight genes (*BmWnt1*, *BmWnt6*, *BmWnt9*, and *BmWnt10*) distributed as a cluster on chromosome 4; *BmWntA*, *BmWnt5*, and *BmWnt7* were concentrated on chromosome 28; while *BmWnt11* existed on chromosome 15 alone (Figure 4). There are many studies indicating that *Wnt9*-*Wnt1*-*Wnt6*-*Wnt10* is an evolutionarily conserved gene cluster in many species, as all or part of this sequence always occurs in a compact cluster arrangement. To analyze the conserved gene cluster in insects, we investigated the gene localization and direction in the 15 species (Figure 5 and Table S3). The analysis identified two gene cluster patterns in the eight species of Lepidoptera. In *Amyelois transitella*, *Bicyclus anynana*, and *Bombyx mori*, the gene cluster was arranged as 5′-*Wnt9*-*Wnt1*-*Wnt6*-*Wnt10*-3′, and in the other five species, the cluster was arranged inversely as 5′-*Wnt10*-*Wnt6*-*Wnt1*-*Wnt9*-3′. In *Drosophila melanogaster*, *Tribolium castaneum*, and *Anopheles gambiae*, the gene cluster was also arranged as 5′-*Wnt9*-*Wnt1*-*Wnt6*-*Wnt10*-3′, but the direction of *Wnt6* in *Drosophila* and the *Wnt9* is opposite to that of the Lepidopteran species. *Apis mellifera* has lost *Wnt9*, and the remaining genes are arranged as 5′-*Wnt1*-*Wnt6*-*Wnt10*-3′. *Acyrthosiphon pisum* has lost three genes, retaining only *Wnt1*. In the two mammals, *Wnt1*-*Wnt10b* and *Wnt6*-*Wnt10a* were located on two different chromosomes, and the direction of *Wnt1* and *Wnt10b* in the two mammals is the opposite.

### 2.4. Embryonic Expression Profile of the BmWnt Genes

**Figure 6:**
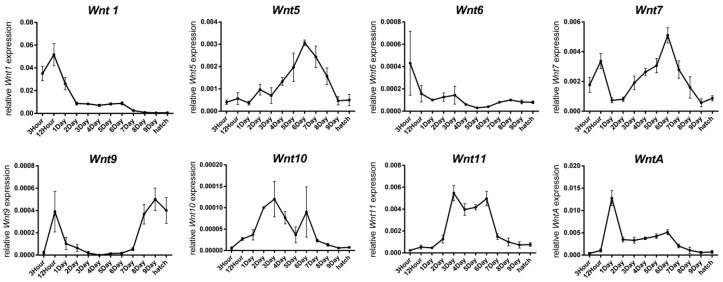
RT-qPCR of the *BmWnt* genes in silkworm embryo. 3Hour: 3 h after egg laying (AEL), 12Hour: 12 h AEL, 1Day–9Day: 1–9 days AEL. Three biological replicates were performed for each sample.

The *Wnt* genes play important roles in embryogenesis of metazoans, including embryonic body axis formation; organogenesis; and cell fate determination, proliferation, migration, and regeneration [31]. To assess the molecular function of the *BmWnt* genes in silkworm embryogenesis, we analyzed expression patterns by RT-qPCR. The *Wnt* gene family members are expressed successively through the development of the embryo (Figure 6). The earliest expression genes are *Wnt1*, *Wnt7*, and *Wnt9,* which are expressed at relatively high levels at 12 h after egg laying (AEL). Then, *Wnt10* and *Wnt11* are expressed later, with expression peaks at three days AEL. The next important developmental stage is six days AEL, when *Wnt5*, *Wnt7*, *Wnt10*, and *Wnt11* are highly expressed. *Wnt9* is expressed at a relatively high level during late stages of embryonic development. Subsets of *Wnt* genes exhibit similar expression patterns, such as *Wnt1* and *WntA* or *Wnt10* and *Wnt11*, suggesting that the genes may have functional similarities and/or synergistic effects in silkworm embryogenesis. The results revealed that *Wnt* genes are expressed in all stages through embryonic development, and different genes are significantly up-regulated at different developmental stages, suggesting that different *Wnt* genes function in different stages of embryonic development.

### 2.5. Different Tissue Expression Profiles of the BmWnt Genes

**Figure 7:**
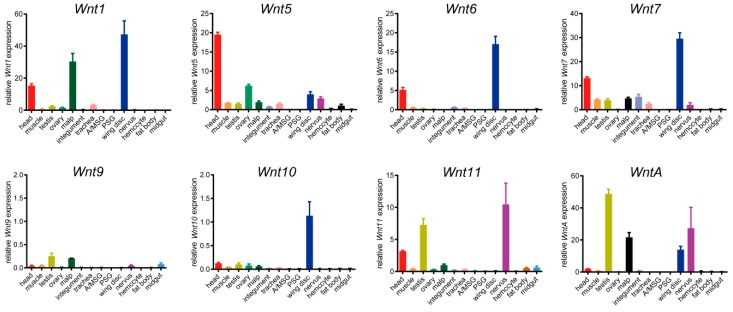
Different tissue expression profiles of the *BmWnt* genes. Multiple larval tissues of the silkworm were collected on the third day of the fifth instar. Differently colored columns represent different tissues. malp: malpighian tubules; A/MSG: anterior/median silk gland; PSG: posterior silk gland.

To determine the spatial expression of *BmWnt* genes, we characterized transcriptional profiles in 14 tissues on the third day of the fifth instar of larval silkworm by RT-qPCR. This stage is a high-food period during which silkworm larvae undergo rapid growth and during which the tissues are in the peak stages of development. The *BmWnt* genes were expressed at low levels in most tissues; high expression levels were present in the head, testis, ovary, wing disc, and nervous system. There were five *Wnt* genes that were relatively highly expressed in the head: *Wnt1*, *Wnt5*, *Wnt6*, *Wnt7*, and *Wnt11*. Most of the *BmWnt* genes were expressed in the wing discs, among which *Wnt1*, *Wnt6*, and *Wnt7* had significantly high expression; *Wnt5*, *Wnt10*, and *WntA* had relatively lower levels of expression, while *Wnt9* and *Wnt11* were not expressed in the wing discs. Several genes may be involved in reproductive organ development; *Wnt5* was expressed in ovary, while *Wnt11* and *WntA* were expressed in testis. In addition, *Wnt11* and *WntA* were highly expressed in the nervous system, and *Wnt1* and *WntA* were highly expressed in malpighian tubules (Figure 7).

## 3. Discussion

We identified eight *Wnt* genes (*Wnt1*, *Wnt5*–*Wnt7*, *Wnt9*–*Wnt11*, and *WntA*) in the silkworm and obtained the same results in seven other species of Lepidoptera, suggesting that the number of *Wnt* genes in Lepidoptera is evolutionarily conserved. According to the Signal 4.1 server’s algorithm, six of the eight BmWnts contain an identifiable signal sequence, with BmWnt7 and BmWnt10 apparently lacking this motif (Figure 1). An analysis of predicted Wnt10 proteins from 14 other species revealed identifiable signal sequences in *Tribolium castanenum* and two mammalian species. A predicted signal peptide was only found in the Wnt7 protein of *Acyrthosiphon pisum*, *Anopheles gambiae*, and *Drosophila melanogaster*. There were other Wnt proteins in different species that have no predicted signal peptides (Table S2). It should be noted that bioinformatic prediction of signaling motifs is limited, and the possibility exists that these proteins contain “cryptic” signal sequences that cannot be recognized by the specific software employed. Additional biochemical/cell biological experiments will be needed to determine whether these are non-secreted or non-canonically secreted Wnts.

The *Wnt* family is novel and conserved in metazoans, being present in animals from sponges to humans [32]. An ancient metazoan is considered to have possessed the diverse 13-member repertoire of *Wnt* genes according to previous studies [19,20]. *Wnt* duplications have occurred in vertebrates and a few species of arthropods, while some *Wnt* subfamilies have been lost in insects during the evolutionary time [33,34]. The number of *Wnt* genes in Lepidoptera is less than that in *Tribolium castaneum* and greater than the numbers in *Drosophila melanogaster*, *Anopheles gambiae*, *Acyrthosiphon pisum*, and *Apis mellifera*, suggesting greater dynamic genome evolution in Lepidoptera than in *Tribolium castaneum* but less than in the other analyzed insects.

We investigated the gene localization and direction of the conserved gene cluster; interestingly, we found that there were two patterns present in the cluster in the eight species of Lepidoptera. The 5′-*Wnt9*-*Wnt1*-*Wnt6*-*Wnt10*-3′ and inverse pattern 5′-*Wnt10*-*Wnt6*-*Wnt1*-*Wnt9*-3′ were both identified in butterflies and moths. The results suggest that genome rearrangements occurred before the divergence of these two classes of Lepidoptera and that both cluster patterns existed in ancient Lepidoptera. In addition, the loss of some genes may be ancestral, such as *Wnt2*, *Wnt3*, and *Wnt4*, which are absent in all insects. In contrast, other losses appear to be more recent, characterized by occurring in specific orders, such as *Wnt6* loss in *Acyrthosiphon pisum*, *Wnt11* loss in *Anopheles gambiae* and *Apis mellifera*, and *WntA* loss in *Drosophila melanogaster*.

*Wnt1* is the most extensively studied *Wnt* gene, and it possesses many essential functions, including the development of the trachea, mesoderm, central nervous system (CNS), eye, imaginal disc, appendages, and wing spots of *Drosophila* [23]. The most important known function of *Wnt1* is in segment polarity in embryogenesis. *Wnt1* is expressed at the blastoderm and germband stages during embryogenesis in *Drosophila* [35,36] and *Tribolium* [37], corresponding to the stage at 20 h AEL of the silkworm. Previous studies had shown that the expression of *Wnt1* in the silkworm embryo is segmentally iterated, suggesting its conserved segment polarity function in the silkworm; loss-of-function analysis based on the CRISPR/Cas9 system revealed that *Wnt1* is necessary during *Bombyx* embryogenesis [27,38]. Here, we found that *Wnt7*, *Wnt9*, and *WntA* also have peaks of expression in early embryonic development resembling that of *Wnt1* (Figure 6). Studies in *Drosophila* had shown that over-expression of *Wnt9* causes segmental patterning defects, suggesting that this gene is involved in segmentation [39,40]; however, *Wnt7* does not appear to be involved in *Drosophila* segmentation, though it is expressed in a segmentally iterated pattern [41]. Thus, whether these simultaneously expressed genes work together or possess functional redundancy in early embryo development of the silkworm needs further experimental verification.

In addition to the important function of *Wnt1*, other *Wnt* genes also play important roles in the development of organisms. *Wnt7* has been shown to be necessary for tracheal development of *Drosophila* [42]. The tracheal development of the silkworm embryo occurs on the sixth day AEL, on which we detected peak expression of *BmWnt7*, suggesting that *Wnt7* may retain the conserved function in silkworm tracheal development.

*Wg*, *Wnt5*, and *Wnt9* had been shown to be involved in regulating axon guidance and synapse formation as key molecules in neurons of *Drosophila* [41,43,44,45,46]. According to our investigation, the situation in the silkworm seems to be different. We detected a small amount of expression of *BmWnt5* and *BmWnt9*; however, *Wnt11* and *WntA* were highly expressed in silkworm nerves (Figure 7), which is consistent with the study on *Litopenaeus vannamei* [47], suggesting that the two genes may have functions in the nerves of Ecdysozoa.

In the larval tissues, *Wnt1*, *Wnt6*, *Wnt7*, *Wnt10*, and *WntA* were detected in wing discs of silkworms (Figure 7). The function of *Wnt1* in wing development is well documented; while *Wnt6* is expressed in wing discs with no effect on wing development in *Drosophila*, this may result from the two genes being regulated by common regulatory elements, as they are only 30 kb apart in *Drosophila* [48]. In addition, *Wnt1* is strongly expressed in wing spots of *Drosophila* [49], while *Wnt1*, *Wnt6*, *Wnt10*, and *WntA* have been shown to participate in color pattern induction in butterflies [50]. Although there are no reports of *Wnt7* involvement in insect wing development, our study detected high expression of *BmWnt7* in silkworm wing discs. Further analysis is needed to determine whether *BmWnt7* is required for wing development.

*Wnt* genes also affect reproduction. In *Drosophila*, *Wnt7* and *Wnt9* mutants show sterile phenotypes [51,52,53]. This study found that *BmWnt11* and *BmWntA* are highly expressed in the testis, suggesting that the *BmWnt* genes may also affect reproduction, but whether this involves different mechanisms than in *Drosophila* needs further experiments to verify. In recent years, although the functions of *Wnt* genes have been subject to analyses, the function and detailed mechanism of *Wnt* genes are not completely clear except for that of the *Wnt1* gene, and less research has been done in insects other than the fruit fly. While our study provides a survey of *BmWnt* expression levels over development and in specific tissues, loss of function analysis, e.g., CRISPR/Cas9-induced mutations, will be needed to further explore *BmWnt* function in this system.

## 4. Materials and Methods

### 4.1. Genome-Wide Identification of Wnt Proteins

The whole-genome protein sequences of the species in this study were downloaded from the following databases: SilkDB (http://silkworm.genomics.org.cn/) [54], Flybase (http://flybase.org/) [55], MonarchBase (http://monarchbase.umassmed.edu/) [56], Butterfly Genome Database (http://www.butterflygenome.org/node/4) [57], VectorBase (https://www.vectorbase.org/organisms/anopheles-gambiae) [58], AphidBase (http://bipaa.genouest.org/is/aphidbase/) [59], Beetlebase (http://beetlebase.org) [60], KONAGAbase (http://dbm.dna.affrc.go.jp/px/) [61], Beebase (http://hymenopteragenome.org/beebase/) [62], lepbase (http://lepbase.org) [63], and NCBI (https://www.ncbi.nlm.nih.gov). The HMM profiles for the Wnt1 domain (PF00110) were downloaded from the Pfam database (http://pfam.sanger.ac.uk/) [29]. The program HMMSEARCH from the HMMER package was used to search the Wnts proteins. The acquired proteins were confirmed by SMART and blast on NCBI.

### 4.2. Chromosomal Distribution and Protein Structure

The “BmWnts distribution map” was displayed by MapChart [64]; gene loci were placed according to their position on the chromosomal information from Silkbase. The domain predictions of BmWnt proteins were performed using the Simple Modular Architecture Research Tool (SMART, available online: http://smart.embl-heidelberg.de/); signal peptide analyses were performed using the Signal 4.1 server (http://www.cbs.dtu.dk/services/SignalP/).

### 4.3. Sequence Alignment and Phylogenetic Analysis

Multiple sequence alignments of the BmWnt proteins were performed by Clustal Omega (https://www.ebi.ac.uk/Tools/msa/clustalo/). The neighbor-joining (N-J) phylogenetic tree was constructed by MEGA 6.0 software with a bootstrap of 1000 replicates. The file was modified in Adobe Illustrator.

### 4.4. RNA Extraction and cDNA Synthesis

Embryos at different developmental stages and different tissues of larvae at the third day of the fifth instar (about 14 days after hatching) were collected in Trizol reagent (Invitrogen, Carlsbad, CA, USA). Total RNA was extracted using a total RNA kit (TaKaRa, Beijing, China) according to the manufacturer’s instructions. RNA quality and concentration were assessed using a Nanodrop 2000 (Thermo Fisher Scientific, Waltham, MA, USA). cDNA samples were synthesized from 1 μg of total RNA using the PrimeScript RT Reagent Kit with gDNA Eraser (TaKaRa, Beijing, China) according to the manufacturer’s instructions.

### 4.5. Real-Time Quantitative PCR

The relative expression of *BmWnt* genes was detected by real-time quantitative PCR (qRT-PCR). Primers were designed by Primer 6 according to the identified gene sequences and are listed in Table S4. qRT-PCR was performed on a CFX96™ Real-Time PCR Detection System (Bio-Rad, Hercules, CA, USA) with a Hieff qPCR SYBR Green Master Mix (YESEN). The reaction system was produced in a volume of 20 μL as follows: 1 μL of cDNA, 10 μL of Hieff qPCR SYBR Green Master Mix, 0.4 μL of forward primer (10 μM), 0.4 μL of reverse primer (10 μM), and ddH_2_O water added up to a final volume of 20 μL. The PCR conditions were as follows: 95 °C for 5 min followed by 40 cycles of 95 °C for 10 s and 60 °C for 30 s. Three biological replicates and three technical replicates were done to eliminate individual differences and technical errors. Relative expression was calculated using the 2^−ΔΔ*C*t^ method [65] with *B. mori eukaryotic translation initiation factor 4A* as a reference.

## 5. Conclusions

In this study, we performed the first identification of the repertoire of *Wnt* genes in Lepidoptera. We obtained eight *Wnt* genes in each of the eight species of Lepidoptera. We analyzed the gene cluster in Lepidoptera and found that there are two contrary patterns (5′-*Wnt9*-*Wnt1*-*Wnt6*-*Wnt10*-3′ and 5′-*Wnt10*-*Wnt6*-*Wnt1*-*Wnt9*-3′) existing in both moths and butterflies. Our research provides valuable insight into *Wnt* gene expression in embryos and multiple tissues of larval silkworms, and the results should be of benefit for further functional studies on the silkworm.

## Figures and Tables

**Table 1:** Summary of *BmWnt* genes.

GeneName	GeneID	Scaff_ID	Probe ID
*BGIBMGA006146*	*BmWnt1*	nscaf2847: 4290086..4300264: +	sw07406
*BGIBMGA013783*	*BmWnt5*	nscaf3097: 574395..598709: +	sw10651
*BGIBMGA006004*	*BmWnt6*	nscaf2847: 4311541..4316713: −	sw10550
*BGIBMGA013981*	*BmWnt7*	nscaf3099: 2430055..2453230: +	none
*BGIBMGA006145*	*BmWnt9*	nscaf2847: 4227852..4228750: +	sw17884
*BGIBMGA006147* *BGIBMGA006148*	*BmWnt10*	nscaf2847: 4406381..4410443: +	sw21982
*BGIBMGA007580*	*BmWnt11*	nscaf2887: 11667287..1177617: +	sw15093
*BGIBMGA013787*	*BmWntA*	nscaf3097: 771657..781653: +	sw13710

The “+” denote the gene on the sense strand, the “−” denote the gene on the antisense strand.

## Supplementary Materials

Supplementary materials can be found at https://www.mdpi.com/1422-0067/20/5/1221/s1.

## Abbreviations

PCP	planar cell polarity
Fzd	Frizzled
LEF1/TCF	T-cell factor/lymphoid enhancing factor
JNK	Jun N-terminal serine/threonine kinase
Rock	Rho-associated kinase
CamKI	Ca^2+^–calmodulin-dependent protein kinase II
PKC	protein kinase C
HMM	Hidden Markov Model
SMART	Simple Modular Architecture Research Tool
AEL	after egg laying
